# Benchmarking a Molecular Flake Model on the Road to Programmable Graphene-Based Single-Atom Catalysts

**DOI:** 10.1021/acs.jpcc.3c07681

**Published:** 2024-02-13

**Authors:** Colin Gallagher, Wali Siddiqui, Tyler Arnold, Carmen Cheng, Eric Su, Qing Zhao

**Affiliations:** Department of Chemical Engineering, Northeastern University, Boston, Massachusetts 02115, United States

## Abstract

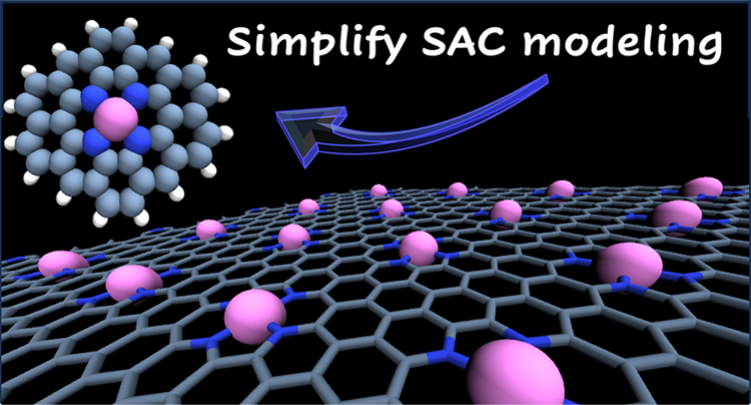

Single-atom catalysts (SACs) of embedding an active metal in nitrogen-doped graphene are emergent catalytic materials in various applications. The rational design of efficient SACs necessitates an electronic and mechanistic understanding of those materials with reliable quantum mechanical simulations. Conventional computational methods of modeling SACs involve using an infinite slab model with periodic boundary condition, limiting to the selection of generalized gradient approximations as the exchange correlation (XC) functional within density functional theory (DFT). However, these DFT approximations suffer from electron self-interaction error and delocalization error, leading to errors in predicted charge-transfer energetics. An alternative strategy is using a molecular flake model, which carved out the important catalytic center by cleaving C–C bonds and employing a hydrogen capping scheme to saturate the innocent dangling bonds at the molecular boundary. By doing so, we can afford more accurate hybrid XC functionals, or even high-level correlated wavefunction theory, to study those materials. In this work, we compared the structural, electronic, and catalytic properties of SACs simulated using molecular flake models and periodic slab models with first-row transition metals as the active sites. Molecular flake models successfully reproduced structural properties, including both global distortion and local metal-coordination environment, as well as electronic properties, including spin magnetic moments and metal partial charges, for all transition metals studied. In addition, we calculated CO binding strength as a descriptor for electrochemical CO_2_ reduction reactivity and noted qualitatively similar trends between two models. Using the computationally efficient molecular flake models, we investigated the effect of tuning Hartree–Fock exchange in a global hybrid functional on the CO binding strength and observed system-dependent sensitivities. Overall, our calculations provide valuable insights into the development of accurate and efficient computational tools to simulate SACs.

## I Introduction

Heterogeneous single-atom catalysts (SACs) that contain isolated single metal atoms coordinated with non-metal atoms (typically with nitrogen or N) embedded on a graphene support are emergent catalytic materials.^1−3^ SACs possess well-defined active sites and can maximize the atom utilization, which is particularly crucial for expensive and rare noble metals.^4−7^ More importantly, SACs exhibit tunable and scalable catalytic reactivities due to their unique atomic and electronic structures, making them active and selective catalysts in various applications, ranging from oxygen reduction reaction,^8−10^ hydrogen evolution reaction,^11−13^ and ammonia electrosynthesis^14,15^ to carbon dioxide (CO_2_) reduction reaction (CO_2_RR),^16−19^ among many others.^1,2^

The rational design of SACs, particularly with respect to controlling activity and selectivity for a specific reaction, necessitates a fundamental understanding of reaction mechanisms and often involves the discovery of structure–property relationships and descriptors correlated with reactivities, typically performed with density functional theory (DFT) calculations. Here, we use electrochemical CO_2_RR as an example to show recent advances in theoretical investigations on SACs. For example, Vijay et al. elucidated reaction mechanisms of CO_2_RR to carbon monoxide (CO) on iron and nickel embedded in nitrogen-doped graphene (Fe–N–C and Ni–N–C), found that either CO_2_ adsorption or proton-coupled electron transfer to form COOH* could be the rate-limiting step,^18^ and revealed the effect of dipole-field interactions in the overall activity.^20^ Zhao and Liu performed *ab initio* molecular dynamics simulations to unravel reaction mechanisms of CO_2_RR to CO on Ni–N–C SACs in the presence of a full explicit description of solvent, explaining its high selectivity over the hydrogen evolution reaction.^21^ Brimley et al. applied grand-canonical DFT to simulate reaction mechanisms of CO_2_RR to CO on M–N–C (M = 3d transition metals) with applied potentials explicitly considered and found that Zn–N–C is particularly active at low and moderate applied potentials.^22^ Following the mechanistic analysis, Karmodak et al. screened multiple SACs (M–N–C, M = 3d transition metals) and dual metal sites SACs (Fe−M–N–C, M = 3d transition metals) using binding strength of CO_2_* and COOH* as descriptors for electrochemical CO_2_RR activity and identified four candidates, Cr–N–C, Mn–N–C, Fe–N–C, and Co–N–C, as potential catalysts for CO_2_RR.^23^ Wang et al. investigated various M–N–C SACs and axial O atom-promoted (M–NO–C) SACs (M = 3d–5d transition metals) as electrocatalysts for CO_2_RR to methane and developed a descriptor based on intrinsic properties of materials to correlate with the catalytic activity for future screening purposes.^24^

All of the above-mentioned computational efforts to study SACs were performed within the DFT level of theory using a slab model with periodic boundary condition and generalized gradient approximations (GGA) to the exchange correlation (XC) functional (i.e., Perdew–Burke–Ernzerhof or PBE,^25^ revised Perdew–Burke–Ernzerhof or RPBE,^26^ and Bayesian error estimation functional or BEEF^27^) or at most a subsequent hybrid XC functional (i.e., Heyd–Scuseria–Ernzerhof or HSE06^28^) to refine the electronic structures. When SACs embedded in doped graphene are simulated, a periodic slab model is typically used to approximate the extended nature of those materials. Periodic boundary conditions limit the selection of DFT approximations to GGA functionals since hybrid functionals with exact exchange incorporated are extremely expensive in plane-wave periodic DFT calculations. Unfortunately, these GGA functionals in DFT are plagued by both one- and many-electron self-interaction errors and a lack of a derivative discontinuity, also referred to as energetic delocalization error.^29−33^ This leads to well-known errors in describing charge-transfer processes, critical for simulating electronic properties^34,35^ and catalytic activities^36−40^ in heterogeneous catalysis. Alternatively, one can use a finite molecular flake model, which carves out the important catalytic center (i.e., metal center, N dopants, and several carbon atoms in the graphene) by cleaving C–C bonds and employing a hydrogen capping scheme to saturate the innocent dangling bonds at the molecular boundary to replace the periodic slab model. By doing so, one can afford DFT calculations with hybrid functionals or even high-level correlated wavefunction theory which suffers less or even no delocalization errors. Jia and Kulik applied molecular flake models when they simulated iron embedded in doped graphene SACs for methane to methanol transformation and investigated the roles of dopants in structural and catalytic properties.^41^ However, the extent to which molecular flake models can reproduce predictions of SACs simulated by using periodic slab models remains unclear.

In this work, we benchmarked structural, electronic, and catalytic properties of SACs simulated by using molecular flake models with those modeled with conventional periodic slab models. We focused on 3d transition metals embedded in N-doped graphene (M–N–C, M = Sc–Zn). First, we benchmarked the geometries and electronic structures predicted by two models. We then compared the CO binding strength as a descriptor for electrochemical CO_2_RR reactivity between two models. Finally, we investigated the effect of tuning percentage of Hartree–Fock (HF) exchange in a hybrid functional on CO binding strength using the molecular flake model to understand how the DFT delocalization error elimination approach performs on the reactivity of heterogeneous catalysis.

## II Computational Details

### II.I DFT Calculations Using Periodic Slab Models

**Figure 1:**
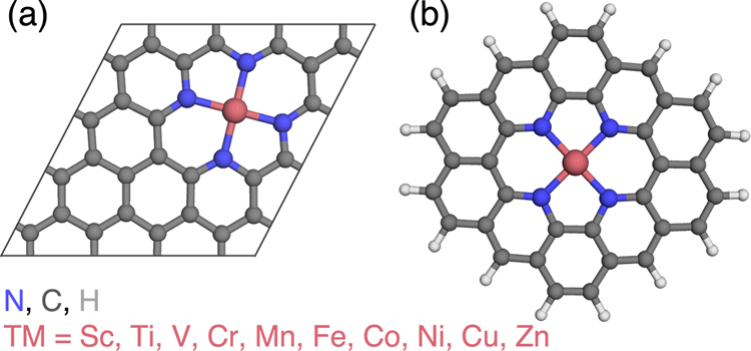
(a) Periodic slab model and (b) molecular flake model with H capping atoms of M–N–C (M = 3d transition metals) SACs. Atoms are colored as follows: transition metal in magenta, N in blue, C in dark gray, and H in light gray.

Spin-polarized periodic DFT calculations were performed using the Vienna Ab initio Simulation Package (VASP)^42,43^ version 6.3.1. All electron, frozen-core, projector augmented-wave (PAW)^44^ method with self-consistently simulated valence electrons of 1s for H, 2s and 2p for C and N, and 4s and 3d for first-row transition metals (Sc, Ti, V, Cr, Mn, Fe, Co, Ni, Cu, Zn) was used. A kinetic energy cutoff of 800 eV was used for the plane-wave basis set. We applied the PBE^25^ XC functional with Grimme’s D3^45,46^ dispersion correction and the Becke–Johnson^47^ damping function in all periodic DFT calculations. To represent the periodic slab model, we employed a unit cell containing one metal atom, four N atoms, and 26 C atoms taken from a previous work^20^ (Figure 1a). The periodic unit cell contained at least 15 Å of vacuum to avoid interactions between the periodic images along the *c*-axis. We fully optimized the unit cell (i.e., optimizing both atomic positions and lattice parameters) until the maximum absolute total force on each atom was smaller than 0.03 eV/Å before adding the CO adsorbate. The adsorbed species was placed on one side of the clean surface with dipole-field energy and potential corrections^48^ applied to cancel the artificial field interaction between the surfaces along the *c*-axis. After addition of the adsorbed species, all atoms in the unit cell were subjected to a subsequent geometry optimization of only atomic positions at fixed lattice parameters of the clean surface using the same convergence threshold. A Γ-point-centered Monkhorst–Pack^49^*k*-point grid of 3 × 3 × 1 was used to sample the Brillouin zone of the unit cell. The selections of the kinetic energy cutoff and *k*-point grid converged the total energies to within 1 meV/atom. Fermi surface smearing with the Methfessel–Paxton scheme^50^ and a smearing width of 0.1 eV was used to sample the Brillouin zone and to aid the electronic structure convergence.

### II.II DFT Calculations Using Molecular Flake Models

DFT calculations using molecular flake models were carried out with TeraChem version 1.9.^51,52^ We used the composite LACVP* basis set, which corresponds to a LANL2DZ effective core potential^53,54^ basis for 3*d* transition metals and the 6-31G* basis for N, C, and H atoms. We applied the same PBE^25^ XC functional to benchmark the effectiveness of using molecular flake models to describe the periodic systems. All possible spin states of each M–N–C SAC were considered using the PBE functional. Singlet spin-state calculations were performed in a spin-restricted formalism, while other spin-state calculations were carried out in a spin-unrestricted formalism. In addition, we employed a modified PBE0^55^ (modPBE0) hybrid XC functional to investigate the effect of incorporating exact exchange on predicting CO binding energies by tuning the percentage of HF exchange from as low as 0% (*a*_HF_ = 0.0 corresponding to a pure PBE GGA functional) to as high as 50% HF (*a*_HF_ = 0.5) exchange in increments of 10%
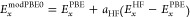
in which *a*_HF_ represents the percentage of HF exchange, and *E*_*x*_^PBE^ and *E*_*x*_^HF^ are the energies of using PBE exchange functional and HF exchange functional, respectively. The original PBE0^55^ functional contains 25% HF exchange (*a*_HF_ = 0.25). We created the molecular flake model on top of the initial periodic slab model by cleaving C–C bonds and capping boundary C atoms with H atoms to saturate the dangling bonds, resulting in a MN_4_C_36_H_16_ compound (Figure 1b). We performed geometry optimizations on the molecular structures using the default L-BFGS optimizer in TeraChem with default convergence thresholds of 4.5 × 10^–4^ hartree/bohr for the maximum gradient and 1.0 × 10^–6^ hartree for the change in energy.

## III Results and Discussion

### III.I Structural Properties Predicted by Periodic and Molecular Models

**Figure 2:**
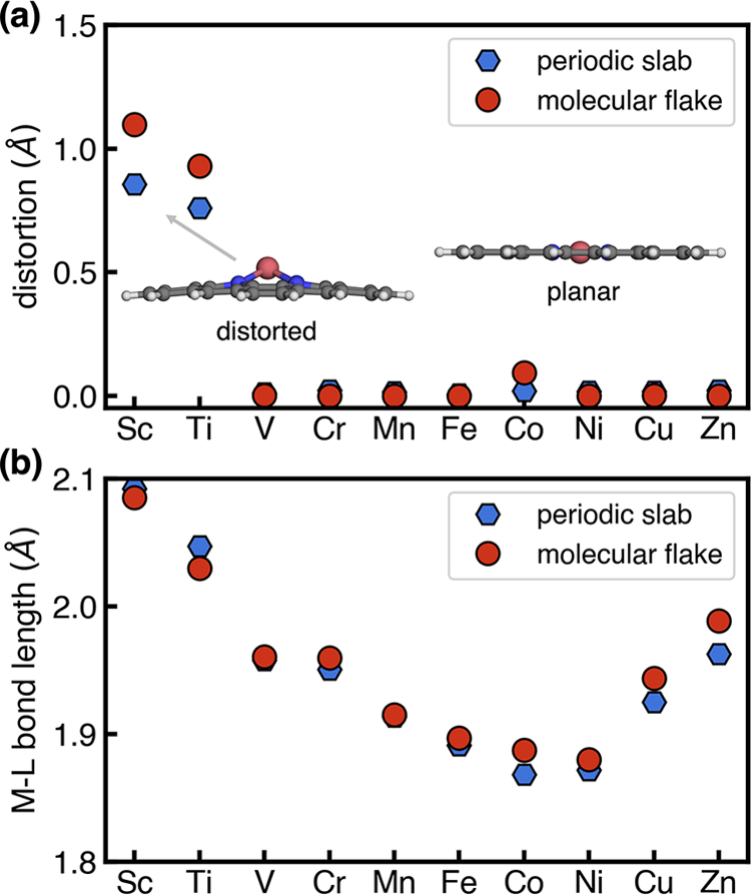
(a) Structural distortions and (b) average bond lengths between metal centers and N dopants of SACs predicted using the PBE functional with a periodic slab model (blue hexagons) or a molecular flake model (red circles). Distortions are measured by calculating the distance between metal center and the center of mass of SAC structure along the *z*-direction. A representative distorted molecular structure and a representative planar molecular structure are shown in the insets.

After full geometry optimizations with periodic boundary conditions, we observed that most M–N–C SACs remain planar, whereas two SACs containing early transition metals (i.e., Sc and Ti) are distorted, with metal centers moving away from the graphene plane. Molecular flake models capped with boundary H atoms successfully reproduced the out-of-plane distortion within Sc–N–C and Ti–N–C, as well as the planar structures of other SACs. To quantify the movement of metal centers and out-of-plane distortion observed in SACs containing early transition metals, we measured the distance between metal center and center of mass of SACs along the *z*-direction (Figure 2a). Sc–N–C and Ti–N–C simulated with both structural models exhibit large distortion distances (periodic slab model: 0.86 Å for Sc–N–C and 0.76 Å for Ti–N–C; molecular flake model: 1.10 Å for Sc–N–C and 0.93 Å for Ti–N–C), while the remaining SACs exhibit consistent negligible distortion distances (i.e., less than 0.10 Å). Since molecular flake models cannot enforce symmetry and rigidity in the metal-coordinating environment as in calculations with periodic boundary conditions, they predict slightly stronger distortions and exhibit curvatures in disordered SACs. To quantify whether H capping atoms were maintained in the same plane as graphene, we measured the distance between the centers of mass of C and H atoms. Only distorted surfaces (Sc–N–C and Ti–N–C) with slight graphene curvature exhibited deviations of ∼0.20 Å with all others smaller than 0.01 Å, confirming that H capping atoms maintained in the same plane as graphene in those molecular analogies and indicating those H atoms properly approximating the nature of extended graphene.

In addition to the overall structural distortion, we also compared the metal–ligand (i.e., N dopant) bond lengths between periodic boundary calculations and molecular flake models. Consistent with the observations of successfully predicting disordered SACs, molecular flake models again reproduced the average metal–ligand bond distances for all M–N–C SACs with differences less than 0.03 Å (Figure 2b). We observed a general trend that molecular flake models slightly underestimate bond lengths for distorted SACs (i.e., Sc–N–C and Ti–N–C), whereas they slightly overestimate bond lengths for planar SACs compared with those predicted with periodic boundary conditions. Thus, in terms of structural properties, we can expect that molecular flake models can reproduce both global distortion and the local metal-local environment of SACs.

### III.II Electronic Properties Predicted by Periodic and Molecular Models

We next compared the magnetic property, i.e., spin magnetic moment, on the dispersed metal atom in SAC predicted by using a periodic slab model and a molecular flake model. Within periodic calculations, we focus on the total magnetization, corresponding to the number of unpaired electrons, i.e., difference between the number of spin-up and spin-down electrons, in the unit cell. Since we used a finite k-point grid to sample the Brillouin zone and added smearing to aid in convergence, orbitals around the Fermi level are partially occupied, leading to a fractional number of unpaired electrons in those periodic calculations. While for molecular systems, the number of unpaired electrons is defined as the spin multiplicity of the molecule, and only integer values are realistic. Total magnetizations in periodic calculations and the preferred spin states in molecular calculations are summarized in Table 1. For middle-row and late transition metals (V, Cr, Mn, Fe, Co, Ni, Cu, and Zn), the predicted number of unpaired electrons is both qualitatively and quantitatively consistent across using the two different models. For the early transition metals, Sc and Ti, the outer 3d shells possess one and two electrons, respectively, indicating a possible spin state of doublet for Sc and singlet or triplet for Ti. Though periodic calculations predict slightly less unpaired electrons than molecular calculations due to the partial occupations of orbitals, they result in consistent favorability over doublet and triplet spin states for Sc and Ti. We verified for both models that the unpaired electrons concentrate on the 3d orbitals of the metal atoms, as expected. Again, evaluating spin magnetic models demonstrates the feasibility of cleaving the C–C bond and capping dangling bonds with hydrogen atoms in a molecular flake structure to simulate periodic SACs.

**Table 1:** Total Magnetizations and Spin States of SACs Evaluated by the PBE Functional Using the Periodic Slab Model and Molecular Flake Model, Respectively

	slab	molecule
Sc	0.30	doublet
Ti	1.57	triplet
V	2.92	quartet
Cr	4.02	quintet
Mn	3.00	quartet
Fe	1.99	triplet
Co	0.95	doublet
Ni	0.00	singlet
Cu	1.05	doublet
Zn	0.00	singlet

**Figure 3:**
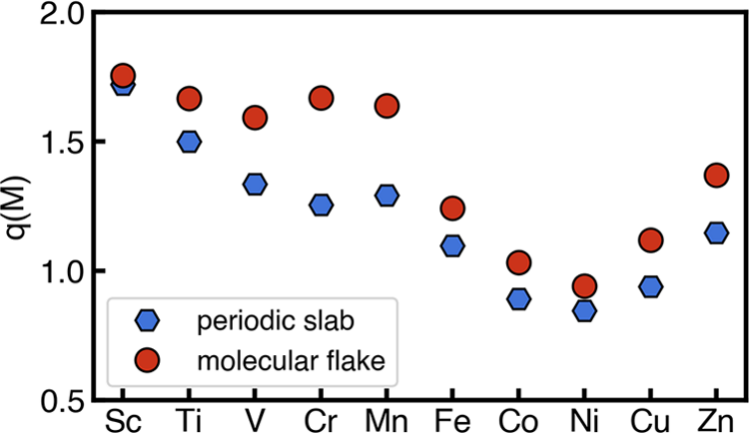
Transition metal partial charge in *e* predicted by using a periodic slab model (red circles) and a molecular flake model (blue hexagons) for SACs.

In addition to the magnetic moments, we also compared the effects on the metal partial charges. For the two structural models, we adopted the same electron density partition method, a real-space Bader atomic charges analysis, using the BADER program.^56^ We generated electron density cube file using Multiwfn^57^ before Bader analysis for the molecular flake calculations. Overall, trends of first decreasing metal partial charge (i.e., from Sc to Ni) and then increasing metal partial charge (i.e., from Ni to Zn) with increasing *d*-filling are consistent across the two models (Figure 3). We observed consistent overestimations of metal partial charges with small differences in absolute values for Sc, Ti, Fe, Co, Ni, Cu, and Zn (<0.2*e*) and large differences for V, Cr, and Mn (0.2–0.4*e*) when performing molecular calculations. Those differences might not originate from cleaving the C–C bond and using a hydrogen capping scheme, since previous studies showed that partial charge predictions are very sensitive to both the electron density partitioning scheme^58^ and the selection of basis set.^34^

### III.III CO Binding Strength as a Descriptor for Electrochemical CO_2_RR Reactivity

**Figure 4:**
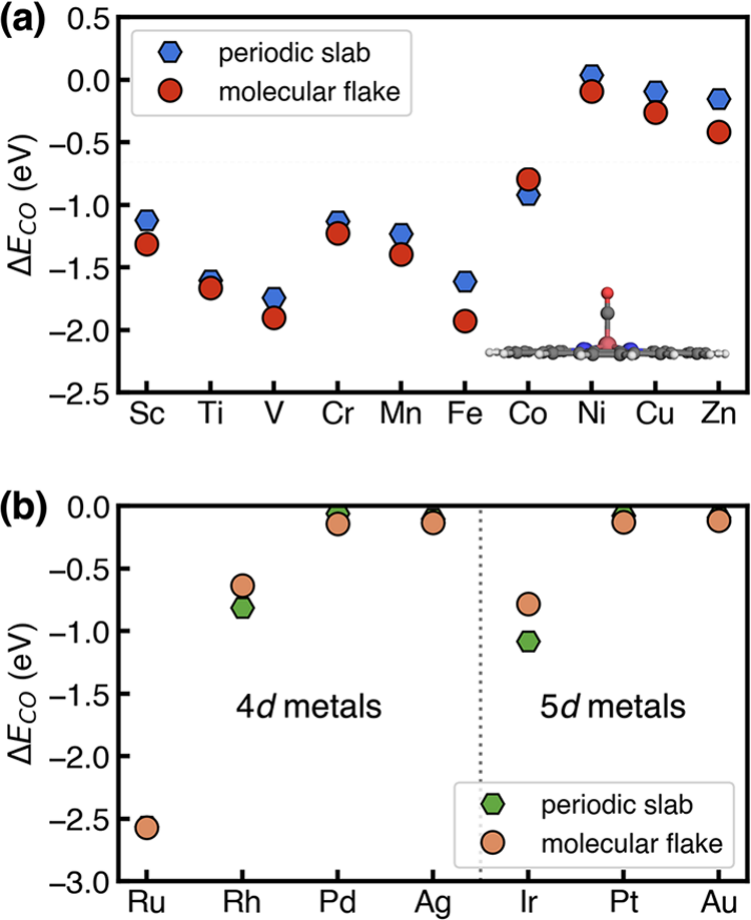
CO adsorption energies in eV on SACs with (a) 3d transition metals and (b) 4d or 5d transition metals as active sites predicted using a periodic slab model (red circles) and a molecular flake model (blue hexagons). A representative CO-adsorbed structure simulated using the molecular flake model is shown in the insets.

The dominant paradigm for catalyst design today is based on the Sabatier principle that the most active catalysts are those that interact with key reaction intermediates neither too strongly nor too weakly.^59^ For electrochemical CO_2_RR, CO binding strength was identified as an effective descriptor to predict catalyst activity since it correlates well with the activation barrier of the rate-limiting step^60^ and thus has been used to design active and selective CO_2_RR electrocatalysts.^61^ Here, we compared CO adsorption energies on SACs with those of CO adsorbed on active metal centers across using conventional periodic slab calculations and simplified molecular flake models. Overall, molecular flake models qualitatively reproduced the CO adsorption energies with a reasonable quantitative accuracy compared with periodic calculations (Figure 4a). The largest differences observed are for Fe–N–C (0.32 eV) and Zn–N–C (0.27 eV), while all other SACs exhibit small differences of less than 0.2 eV. We also noted that molecular flake models consistently predicted stronger CO binding strength compared with periodic slab calculations with the only exception of Co–N–C. Regarding the activity for the CO_2_RR, both methods predict relatively strong binding strength for early and middle-row transition metals and weak binding strength for late transition metals (Ni–N–C, Cu–N–C, and Zn–N–C). These observations are consistent with experiments that all three late transition metals can generate CO with high Faradaic efficiencies,^62^ especially Ni–N–C showing an extremely high selectivity toward producing CO.^17^ CO binding strength on other SACs might be too strong to desorb from the surface as well as to be further hydrogenated toward generating hydrocarbons. In all of the above molecular calculations, we used a molecular analogy of MN_4_C_36_H_16_ to simulate SACs. To determine the size effects of graphene flakes on SAC predictions, we constructed two larger models, MN_4_C_74_H_22_ and MN_4_C_124_H_28_, with the metal site selected as Co due to its moderate CO binding strength. For the three flake sizes, the predicted CO adsorption energies remain unchanged (0.79 eV for MN_4_C_36_H_16_, 0.80 eV for MN_4_C_74_H_22_ and MN_4_C_124_H_28_).

To investigate the transferability of the observed trends from 3d transition metals to 4d or 5d transition metals, we computed the CO adsorption energies on SACs with 4d (Ru, Rh, Pd, Ag) or 5d (Ir, Pt, Au) metal sites. Those elements were selected due to their broad applications as catalytic materials in energy and sustainability. Similarly, we observed that their molecular analogies resulted in a comparable CO binding strength as those obtained in conventional periodic calculations (Figure 4b), indicating the robustness of using less computationally demanding molecular flake models to predict the catalytic properties of SACs for the purpose of high-throughput screening.

### III.IV Effect of HF Exchange and XC Functionals on Tuning CO Binding Strength

All calculations discussed thus far employed a GGA XC functional, PBE, in both periodic slab models and molecular flake models. However, GGA functionals suffer from delocalization error,^29,30^ typically leading to spurious favorable (i.e., more negative) adsorption energies.^63,64^ Delocalization error has a quantitative energetic definition as the deviation from piecewise linearity with fractional addition or removal of an electron.^65^ GGA functionals exhibit a convex deviation, whereas HF exchange conversely overlocalizes electrons and exhibits a concave deviation.^29,66^ The divergent behaviors between GGA functionals and the HF method motivate the development of hybrid XC functionals with an admixture of HF exchange in GGA XC functionals to recover piecewise linearity. Though hybrid functionals can eliminate delocalization error, whether they disfavor adsorption energies on SACs remains unknown. Unfortunately, performing hybrid functional calculations with periodic boundary conditions, especially in the most widely used plane-wave codes, is usually intractable due to high computational cost.

**Figure 5:**
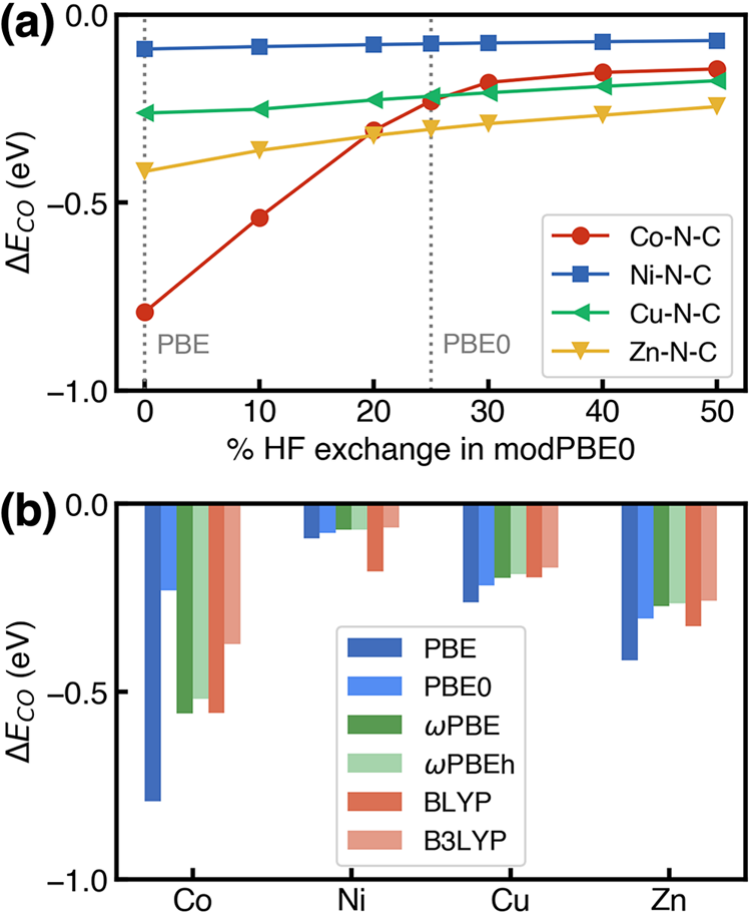
Dependence of CO adsorption energy (a) with percentage of HF exchange in a modified PBE0 hybrid XC functional for M–N–C with M = Co (red circles), Ni (blue squares), Cu (green triangles), and Zn (yellow triangles) and (b) on DFT XC functionals, including PBE (blue), PBE0 (light blue), ωPBEh (green), ωPBE (light green), BLYP (red), and B3LYP (light red) for M–N–C (M = Co, Ni, Cu, Zn) predicted by using molecular flake models. The PBE and PBE0 results are shown with vertical dashed lines in (a).

Instead, we used the molecular flake models to investigate the effect of tuning percentage of HF exchange through a modified PBE0 functional (see the Computational Details section) on predicting CO adsorption energies on M–N–C (Co, Ni, Cu, and Zn) SACs. These four metals were selected since they exhibit moderate (−0.42 eV for Zn–N–C; –0.79 eV for Co–N–C) to weak (−0.09 eV for Ni–N–C; –0.26 eV for Cu–N–C) CO binding strengths at the PBE level, making them hold great promise for catalyzing the CO_2_RR to generate hydrocarbons (Zn–N–C and Co–N–C) or CO (Ni–N–C and Cu–N–C). Using molecular flake models allowed us to fully optimize the geometries by using the more computationally demanding hybrid functionals. Unsurprisingly, we observed that incorporating HF exchange uniformly disfavors CO binding, but with system-dependent sensitivity (Figure 5a). The sensitivity to HF exchange of the CO adsorption energy predictions is high for Co–N–C, changing the binding strength from −0.79 eV at 0% HF exchange (i.e., PBE functional) to −0.14 eV at 50% HF exchange, while HF exchange has a smaller effect on the other three SACs with changes in the CO adsorption energies of less than 0.2 eV (0.17 eV for Zn–N–C; 0.09 eV for Cu–N–C; 0.02 eV for Ni–N–C) predicted at 50% HF exchange and 0% HF exchange. Our results are consistent with the work of Wu et al.^67^ that HF exchange tuning has a negligible effect on CO binding strength for Ni–N–C SACs. Interestingly, we observed a clear correlation between the sensitivities and the CO adsorption energy values themselves, with the highest sensitivities for the most strongly bound species (i.e., Co–N–C), indicating that hybrid functionals tend to disfavor adsorption more significantly for strongly bound systems. Different sensitivities to HF exchange alter the trends in predicting the most favorable binding energies: PBE predicts that CO binding most strongly to Co–N–C, while increasing the percentage HF exchange to 25% (i.e., conventional PBE0) predicts that CO is more favorably adsorbed on Zn–N–C. The predicted weaker CO adsorption energies using hybrid functionals indicate that CO might be the electrochemical CO_2_RR products regardless of metals since adsorbed CO will desorb from surfaces before further hydrogenation.

Since incorporation of HF exchange in a modified PBE0 XC functional consistently disfavors CO adsorption on SACs, we next investigated whether these changes applied to other XC functionals. When 20% HF exchange is added to a pure GGA functional (BLYP^68,69^), resulting in a hybrid B3LYP^69,70^ functional, the CO adsorption energies unsurprisingly become less negative regardless of metal centers (Figure 5b), consistent with the observations of using PBE and PBE0. In addition to global hybrids, we also noted that the inclusion of HF exchange only in the long range as implemented in range-separated hybrid functionals, ωPBE^71^ (no short-range exchange, range-separation decay ω = 0.3 bohr^–1^) and ωPBEh^72^ (20% short-range exchange, range-separation decay ω = 0.2 bohr^–1^), successfully disfavors CO binding strength compared with their pure GGA functional, PBE. Therefore, across a wide range of SACs (i.e., M–N–C, where M = Co, Ni, Cu, Zn), incorporation of HF exchange in a GGA functional, even in the long range only, consistently improves the description of surface and adsorption energy.

## IV Conclusions

We benchmarked a molecular flake model, in which we carved out the important catalytic center by cleaving C–C bonds and employing a hydrogen capping scheme to saturate the innocent dangling bonds at the molecular boundary against the conventional computationally expensive periodic slab model in simulating graphene-based SACs. We systematically compared structural, electronic, and catalytic properties of SACs predicted by the two different models and focused on all 3d transition metals to rationalize the effect with d-filling. Interestingly, we observed that the molecular flake models successfully and qualitatively reproduced all properties investigated, including global distortion, the local metal-coordination environment, spin magnetic moments, metal partial charges, and CO binding strength, with reasonable quantitative accuracy, indicating the feasibility of using molecular flake models to simulate SACs. Using computationally efficient molecular models, one can afford to perform DFT calculations with more accurate hybrid XC functionals and even high-level correlated wavefunction theory calculations. As a demonstration, using the molecular flake model, we investigated the effect of exact exchange in a modified hybrid PBE0 functional on tuning the CO binding strength and confirmed that hybrid functionals can successfully eliminate the overbinding problem of GGA XC functionals but with a system-dependent sensitivity to the incorporated HF exchange.

Thus, by comparing two models to simulate SACs, we rigorously confirmed that the computationally efficient molecular flake model accurately reproduced all structural, electronic, and catalytic properties of SACs at the DFT level of theory. However, high-level multireference methods, such as active space configuration interaction coupled with second-order perturbation theory, are more suitable to simulate SACs due to the inherent self-interaction error and single-reference description of the electronic structure within DFT, and thus we are applying such rigorous methods to reinvestigate SAC activity in ongoing work using the validated molecular flake models, which are otherwise infeasible using the conventional periodic calculations. We believe this work to be useful for both theory development and the catalyst design of SACs.
